# ﻿*Ranunculusjiguanshanicus* (Ranunculaceae), a new species from Sichuan, China

**DOI:** 10.3897/phytokeys.219.96266

**Published:** 2023-01-25

**Authors:** Wen-Qun Fei, Qiong Yuan, Qin-Er Yang

**Affiliations:** 1 Key Laboratory of Plant Resources Conservation and Sustainable Utilization, South China Botanical Garden, Chinese Academy of Sciences, Guangzhou 510655, Guangdong, China; 2 University of Chinese Academy of Sciences, Beijing 100049, China; 3 Center of Conservation Biology, Core Botanical Gardens, South China Botanical Garden, Chinese Academy of Sciences, Guangzhou 510655, Guangdong, China

**Keywords:** Asia, buttercups, Ranunculales, *Ranunculusglareosus*, *Ranunculuspegaeus*

## Abstract

*Ranunculusjiguanshanicus* (Ranunculaceae), a new species from Chongzhou in Sichuan province, China, is here described and illustrated. The new species is easily distinguishable from other Chinese members of the genus by an array of characters, including small stature, glabrous and prostrate stems, 3-foliolate leaves with obvious petiolules (3–5 mm long), unequally 3-sected leaflets, lanceolate to linear ultimate leaflet segments, small flowers (5.2–6 mm in diameter), and long styles in the carpels and achenes (ca. 0.8 mm long). A distribution map of this new species is also provided.

## ﻿Introduction

*Ranunculus* L., comprising approximately 600 species, is the largest genus in the Ranunculaceae and is widely distributed in all continents (Tamura 1995; Hörandl et al. 2005; Paun et al. 2005; Hörandl and Emadzade 2012). In China, one of the centers of species diversity of *Ranunculus*, more than 150 species and 30 varieties are currently recognized in the genus (Wang 1995a, b, 1996, 2007, 2008, 2013, 2015, 2016, 2018, 2019a, b, 2022; Yang 2000; Wang and Gilbert 2001; Wang and Liao 2009; Luo and Zhao 2013; Wang and Chen 2015; Wang et al. 2016; Yuan and Yang 2017a, b, c; Zhang et al. 2020; Fei et al. 2022, 2023a, b).

**Figure 1.**
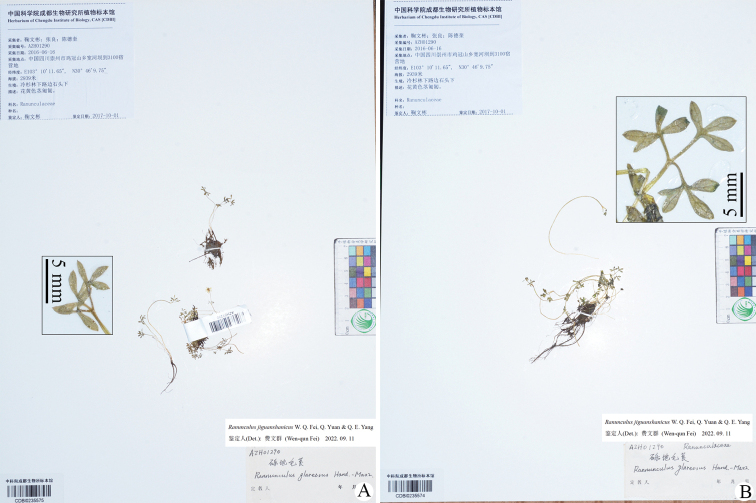
Two specimens of *Ranunculusjiguanshanicus* sp. nov. (**A, B**) previously misidentified as *R.glareosus*. China, Sichuan province, Chongzhou, Anzihe Nature Reserve, Jiguan Shan, 30°46'9.75"N, 103°10'11.65"E, alt. 2939 m, on rocks in fir forest, 16 June 2016, *W.B. Ju*, *L. Zhang & D.K. Chen AZH01290* (CDBI). Insets: leaf blades.

During a survey of herbarium specimens of *Ranunculus* from China for the first author’s Ph.D. dissertation project, one gathering, *W.B. Ju*, *L. Zhang & D.K. Chen AZH01290* (CDBI) (Fig. 1), from Jiguan Shan in the Anzihe Nature Reserve in Chongzhou, Sichuan province, China, caught our attention. This gathering had been previously identified on the determination slips as *R.glareosus* Hand.-Mazz., a species distributed in China’s Qinghai, Sichuan, Xizang (Tibet) and Yunnan (Handel-Mazzetti 1931; Liou 1980; Wang and Gilbert 2001). The plants on the two sheets of the gathering, which have unique leaf morphology and very small flowers, certainly do not belong to *R.glareosus* or any other members of *Ranunculus* currently known for China.

During a botanical expedition to Sichuan from June to July 2022, we successfully discovered a flowering population of this species in early June on Jiguan Shan in Chongzhou, where the gathering *W.B. Ju*, *L. Zhang & D.K. Chen AZH01290* was made. Moreover, we discovered a fruiting population in early July on Xiling Xue Shan in Dayi, a mountain closely adjacent to Jiguan Shan. Based on our observations of living plants in the wild, we confirmed all the diagnostic characters of the new species observed from the herbarium specimens and determined that the gathering and the two populations in question represent a new species. Morphologically, this new species is somewhat similar to *R.pegaeus* Hand.-Mazz., a species occurring in southwestern China (Sichuan, Xizang, Yunnan), India (Sikkim) and Nepal (Handel-Mazzetti 1939; Liou 1980; Wang and Gilbert 2001), but differs by an array of characters. It is described below as *R.jiguanshanicus*.

## ﻿Materials and methods

For morphological comparison, we critically examined specimens or high-resolution specimen images of *Ranunculusglareosus*, *R.jiguanshanicus* and *R.pegaeus* at CDBI, E, KUN, PE, and WU (acronyms according to Thiers 2022). We also observed living plants in one population each in *R.glareosus* (Menyuan in Qinghai province) and *R.pegaeus* (Maoxian in Sichuan province) and those in two populations of *R.jiguanshanicus* (Chongzhou and Dayi in Sichuan province). The morphological description of *R.jiguanshanicus* was based on observations of both herbarium specimens and living plants in the wild.

## ﻿Taxonomy

### ﻿ Ranunculus jiguanshanicus

Taxon classificationPlantaeRanunculalesRanunculaceae

W.Q.Fei, Q.Yuan & Q.E.Yang sp. nov.

2BCA64EF-A813-5D77-97AD-83B236A8591D

urn:lsid:ipni.org:names:77312739-1

**Figure 2.**
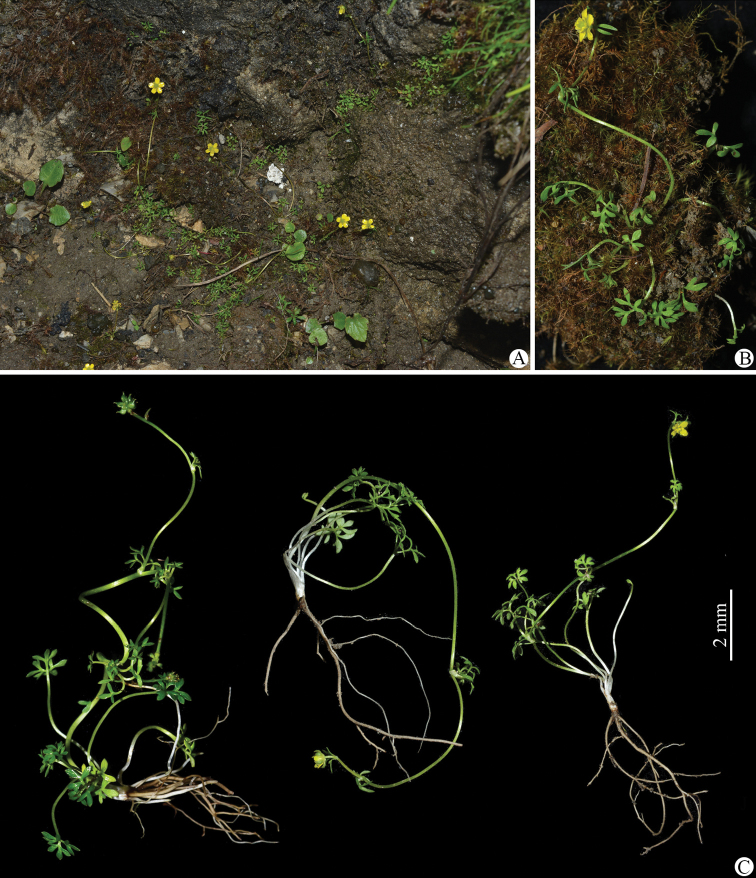
*Ranunculusjiguanshanicus* sp. nov. in the wild **A, B** habitat **C** habit. The left plant (at fruiting stage) in **B** photographed by De-Chang Meng from Xiling Xue Shan in Dayi, Sichuan province, and the right two plants (at flowering stage) photographed by Wen-Qun Fei from Jiguan Shan in the Anzihe Nature Reserve in Chongzhou, Sichuan province.

**Figure 3.**
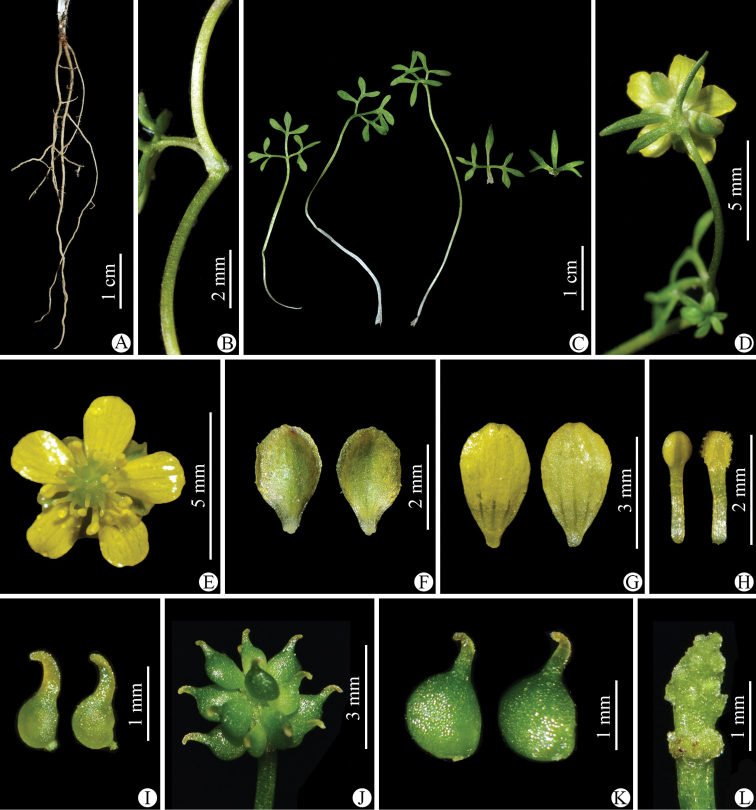
*Ranunculusjiguanshanicus* sp. nov. in the wild **A** roots **B** portion of stem **C** leaves **D** flower (lateral view) **E** flower (top view) **F** sepal (left: abaxial side; right: adaxial side) **G** petal (left: adaxial side; right: abaxial side) **H** stamens **I** carpels **J** aggregate fruit **K** achenes **L** receptacle. **A–H** photographed by Wen-Qun Fei from the population on Jiguan Shan in Chongzhou, Sichuan province and **I–L** photographed by De-Chang Meng from the population on Xiling Xue Shan in Dayi, Sichuan province.

**Figure 4.**
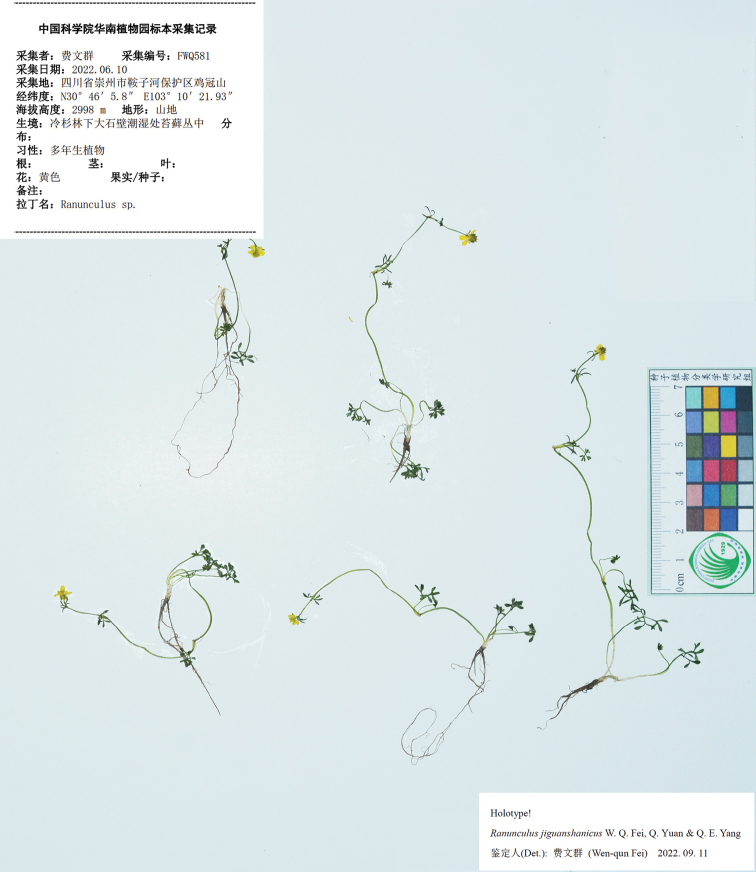
Holotype sheet of *Ranunculusjiguanshanicus* sp. nov.

**Figure 5.**
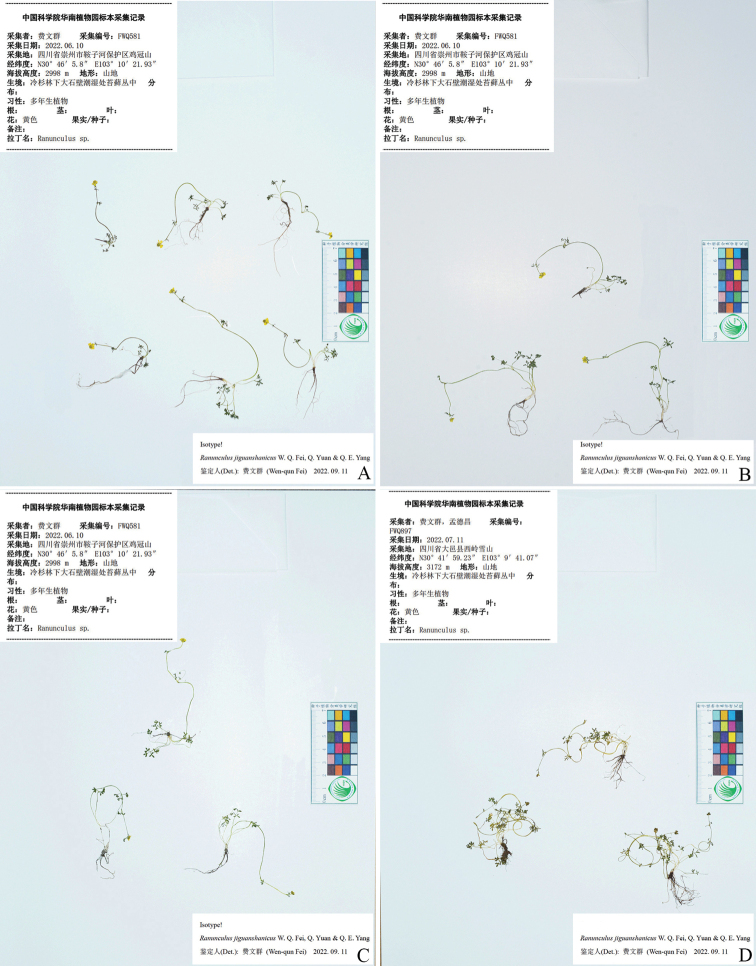
Isotype (**A–C**) and paratype (**D**) sheets of *Ranunculusjiguanshanicus* sp. nov.

Figs 1 , 2 , 3 , 4 , 5

#### Diagnosis.

*Ranunculusjiguanshanicus* is readily distinguishable from all other Chinese species of *Ranunculus* by a unique array of characters, including small stature, glabrous and prostrate stems, 3-foliolate leaves with obvious petiolules (3–5 mm long), unequally 3-sected leaflets, lanceolate to linear ultimate leaflet segments, small flowers (5.2–6 mm in diameter), and long styles in the carpels and achenes (ca. 0.8 mm long).

#### Type.

China. Sichuan province: Chongzhou, Anzihe Nature Reserve, Jiguan Shan, 30°46'5.8"N, 103°10'21.93"E, alt. 2998 m, among moss on rocks or rocky cliffs in moist places in fir forests, 10 June 2022, *W.Q. Fei 581* (holotype: IBSC; isotypes: IBSC, PE).

#### Description.

***Herbs*** perennial, terrestrial or rupicolous. ***Roots*** 2–5, 6–10 cm long, fibrous, slender, slightly thickened at base. ***Stems*** 7–15 cm long, prostrate, glabrous, unbranched to few-branched. ***Basal leaves*** 2–5, 3-foliolate, long-petiolate; petioles 2–4 cm long, glabrous; blades 0.8–1 × 0.8–1.3 cm, suborbicular, thinly chartaceous, adaxially green, abaxially light green, both sides glabrous; leaflets 3, unequally 3-sected, petiolulate, petiolules 3–5 mm long, ultimate leaflet segments 3–4 × 0.8–1.2 mm, narrowly lanceolate to linear, margin entire, apex 1–2-denticulate to 1–2-cleft. ***Lower cauline leaves*** 2–3, similar to basal ones but smaller. ***Upper cauline leaves*** 1–2, 3-foliolate, subsessile or sessile, adaxially glabrous or sparsely puberulous, abaxially glabrous, central leaflet 4.5–5 × 1–1.2 mm, narrowly lanceolate to linear, margin entire, lateral leaflets entire, 1–2-lobate or 2–3-sected, ultimate leaflet segments 3–3.5 × 1–1.2 mm, narrowly lanceolate to linear. ***Inflorescences*** terminal, 1(–2)-flowered. ***Flowers*** 5.2–6 mm in diameter; pedicels 1–2 cm long, glabrous or sparsely puberulous; receptacles ca. 1.2 mm long, clavate, glabrous; sepals 5, 2.2–2.5 × 1.5–1.8 mm, elliptic to obovate, patent, green tinged with yellowish, concave, both sides glabrous; petals 5(–6), 3.2–3.5 × 1.8–2 mm, obovate, yellow, glabrous, apex rounded, nectary pit without a scale, claws ca. 0.4 mm long; stamens 6–8, ca. 2 mm long, filaments ca. 1.5 mm long, narrowly linear, anthers ca. 0.5 mm long, oblong; gynoecium subglobose; carpels 8–12, ovaries ca. 0.8 × 0.6 mm, ovoid, laterally flattened, biconvex, glabrous, styles ca. 0.8 mm long, glabrous, apex recurved. ***Aggregate fruit*** ca. 4 × 4.2 mm, subglobose; achenes ca. 1.2 × 1 mm, widely ovoid, laterally flattened, biconvex, glabrous, styles ca. 0.8 mm long, persistent, glabrous, apex recurved.

#### Etymology.

The specific epithet refers to the type locality of the new species, i.e. Jiguan Shan in the Anzihe Nature Reserve in Chongzhou, Sichuan province, China.

#### Phenology.

Flowering in early June; fruiting at the end of June.

#### Distribution and habitat.

**Figure 6.**
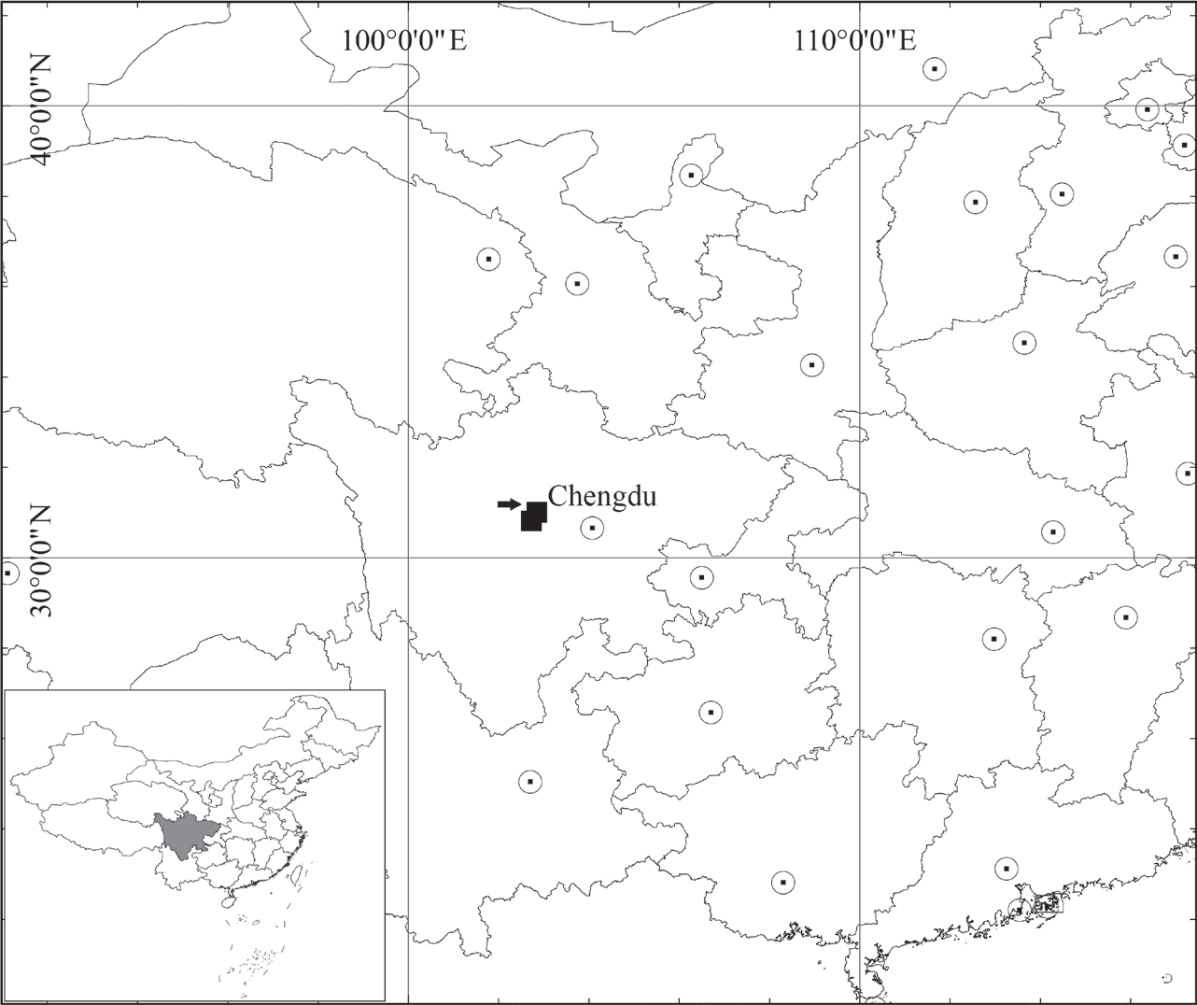
Distribution of *Ranunculusjiguanshanicus* sp. nov. (black square). Arrow indicates the type locality.

*Ranunculusjiguanshanicus* is currently known from its type locality, i.e., Jiguan Shan in the Anzihe Nature Reserve in Chongzhou, and from the closely adjacent Xiling Xue Shan in Dayi, both in Sichuan province, China (Fig. 6). It grows among moss on rocks or rocky cliffs in moist places in fir forests at altitudes of 2900–3150 m above sea level.

#### Conservation status.

*Ranunculusjiguanshanicus* is currently known only from two populations in Sichuan province, China. The Chongzhou population consists of ca. 150 individuals within an area of less than 10 m^2^. The size of the Dayi population remains unknown. The conservation status of *R.jiguanshanicus* should better be categorized as “Data Deficient (DD)” before adequate information of this species is acquired (IUCN Standards and Petitions Committee 2022).

#### Discussion.

*Ranunculusjiguanshanicus* is readily assigned to R.sect.Ranunculus due to its swollen achenes with a distinct beak and receptacles hardly enlarged after anthesis. In his infrageneric classification of the Chinese *Ranunculus*, Wang (1995a, b) placed almost all the alpine species within this section under the name R.sect.Auricomus (Spach) Schur.

**Figure 7.**
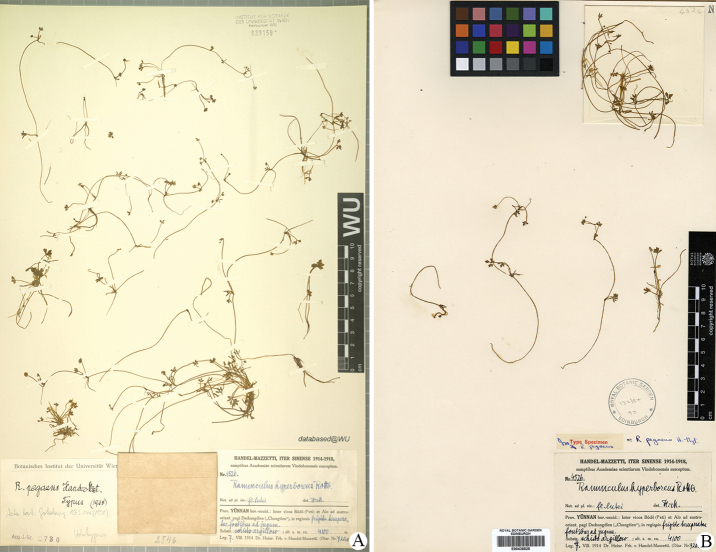
Type sheets (**A, B**) of *Ranunculuspegaeus*.

**Figure 8.**
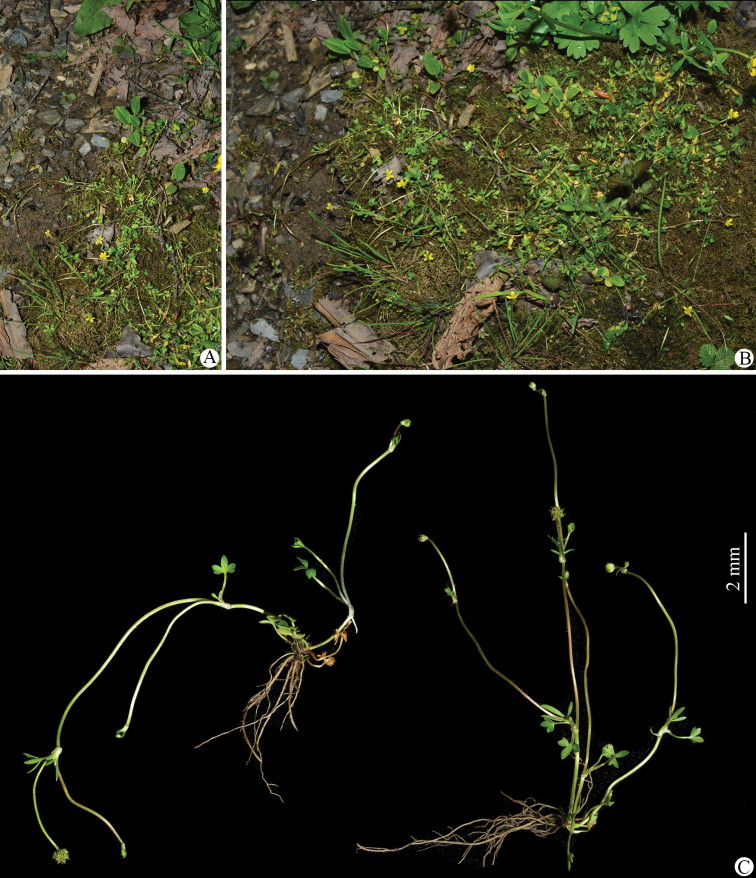
*Ranunculuspegaeus* in the wild (China, Sichuan, Maoxian) **A, B** habitat **C** habit. Photographed by Wen-Qun Fei.

**Figure 9.**
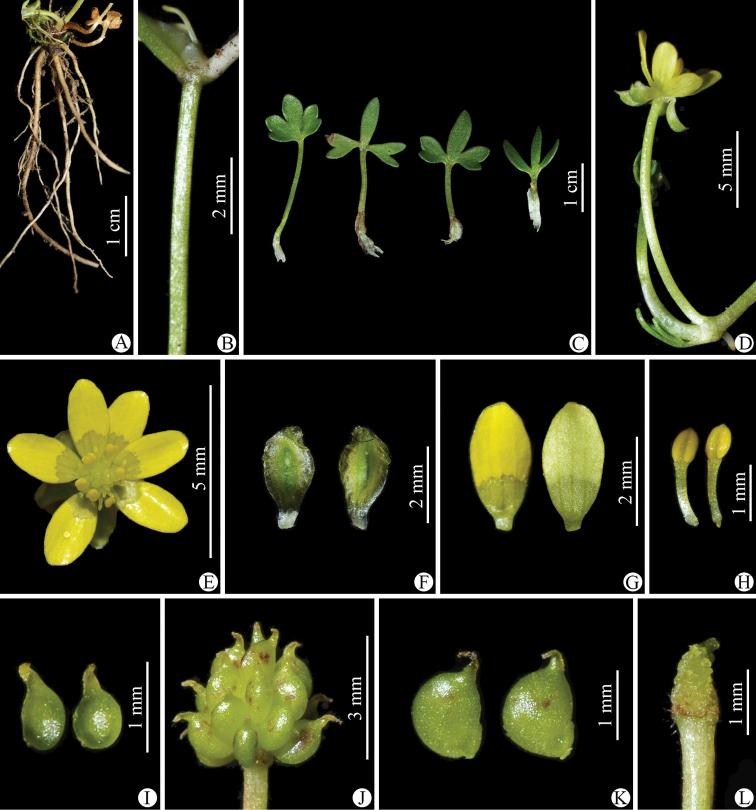
*Ranunculuspegaeus* in the wild (China, Sichuan, Maoxian) **A** roots **B** portion of stem **C** leaves **D** flower (lateral view) **E** flower (top view) **F** sepal (left: abaxial side; right: adaxial side) **G** petal (left: adaxial side; right: abaxial side) **H** stamens **I** carpels **J** aggregate fruit **K** achenes **L** receptacle. Photographed by Wen-Qun Fei.

**Figure 10.**
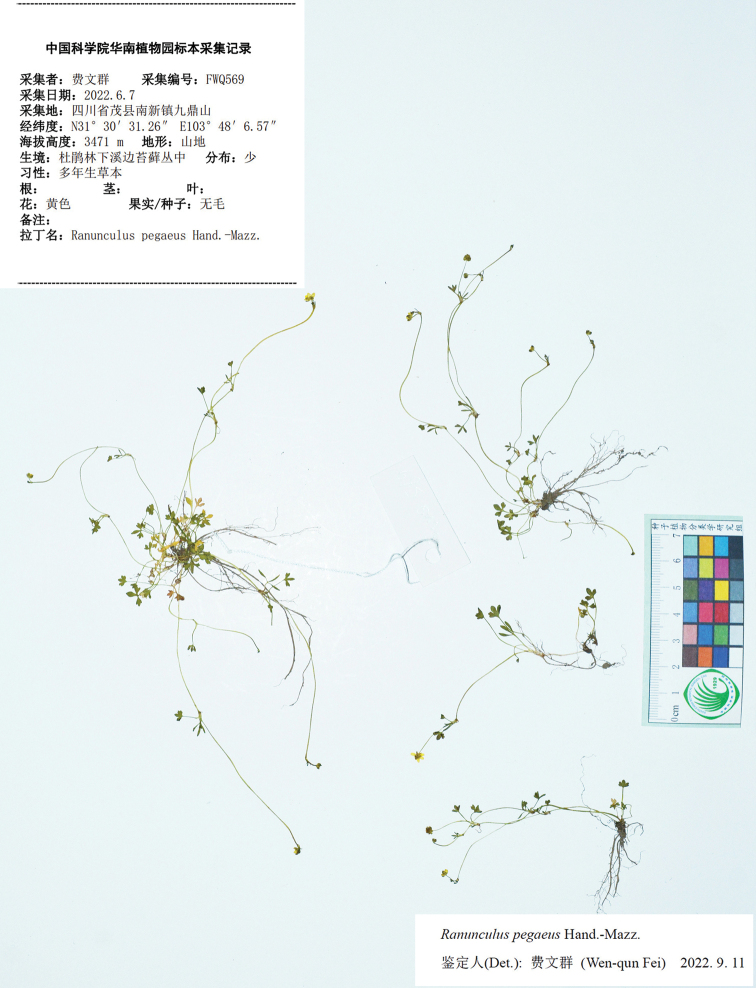
*Ranunculuspegaeus*. China, Sichuan, Maoxian, *W.Q. Fei 569* (IBSC).

Morphologically, *Ranunculusjiguanshanicus* is somewhat similar to *R.pegaeus* (Figs 7–10), also a member of R.sect.Ranunculus, in having prostrate and glabrous stems (Figs 2A, B, 8A, B), small flowers (Figs 3D, 9D), subglobose aggregate fruit (Figs 3I, 9I), and glabrous carpels (Figs 3H, 9H), achenes (Figs 3G, 9G) and receptacles (Figs 3K, 9K). However, it differs by having 3-foliolate leaves with obvious petiolules (3–5 mm long), unequally 3-sected leaflets, lanceolate to linear, entire or 1–2-denticulate to 1–2-cleft ultimate leaflet segments (Fig. 3C), and styles in the carpels and achenes ca. 0.8 mm long (Fig. 3H, G). In *R.pegaeus*, the leaves are 3-partite, 3-sected or 3-foliolate with the central segment/leaflet rhombic or oblong, entire or 3-denticulate and the lateral segments/leaflets obliquely flabellate, entire or unequally 2-cleft (Figs 9C, 10), and styles in the carpels and achenes ca. 0.3 mm long (Fig. 9H, G). A detailed morphological comparison between *R.jiguanshanicus* and *R.pegaeus* is given in Table 1.

**Table 1.** Morphological comparison of *Ranunculusglareosus*, *R.jiguanshanicus* sp. nov. and *R.pegaeus*.

	*R.glareosus*	*R.jiguanshanicus*	*R.pegaeus*
Roots	2‒5, more than 15 cm long	2‒5, 6‒10 cm long	5‒10, 8‒12 cm long
Stems	sparsely puberulous	glabrous	glabrous
Basal leaves	3-sected or 3-foliolate, fleshy, adaxially glabrous or sparsely puberulous, abaxially glabrous, central segment/leaflet ovate or rhombic, entire or 3-lobate, lateral segments/leaflets flabellate, unequally 2-partite	3-foliolate, thinly chartaceous, both sides glabrous, leaflets unequally 3-sected, with ultimate leaf segments narrowly lanceolate to linear, entire or 1‒2-denticulate to 1‒2-cleft	3-partite, 3-sected or 3-foliolate, thinly chartaceous, both sides glabrous, central segment/leaflet rhombic or oblong, entire or 3-denticulate, lateral segments/leaflets obliquely flabellate, entire or unequally 2-cleft
Flowers	terminal, 1‒4, 15‒17 mm in diameter	terminal, 1(‒2), 5.2‒6 mm in diameter	terminal or axillary, 3‒7, 5.5‒8 mm in diameter
Receptacles	3‒5 mm long, clavate, glabrous	ca. 1.2 mm long, clavate, glabrous	ca. 1 mm long, clavate, glabrous
Sepals	adaxially glabrous, abaxially puberulous	both sides glabrous	both sides glabrous
Petals	9‒10 × 7‒8 mm, widely obovate	3.2‒3.5 × 1.8‒2 mm, obovate	3‒3.5 × 1.5‒1.7 mm, obovate
Carpels	20‒35; ovaries ovoid, glabrous; styles ca. 0.2 mm long, straight	8‒12; ovaries ovoid, glabrous; styles ca. 0.8 mm long, apex recurved	18‒22; ovaries ovoid, glabrous; styles ca. 0.3 mm long, apex recurved
Aggregate fruit	ellipsoid	subglobose	subglobose
Achenes	widely ovoid, glabrous, styles ca. 0.2 mm long, straight	widely ovoid, glabrous, styles ca. 0.8 mm long, apex recurved	widely ovoid, glabrous, styles ca. 0.3 mm long, apex recurved

**Figure 11.**
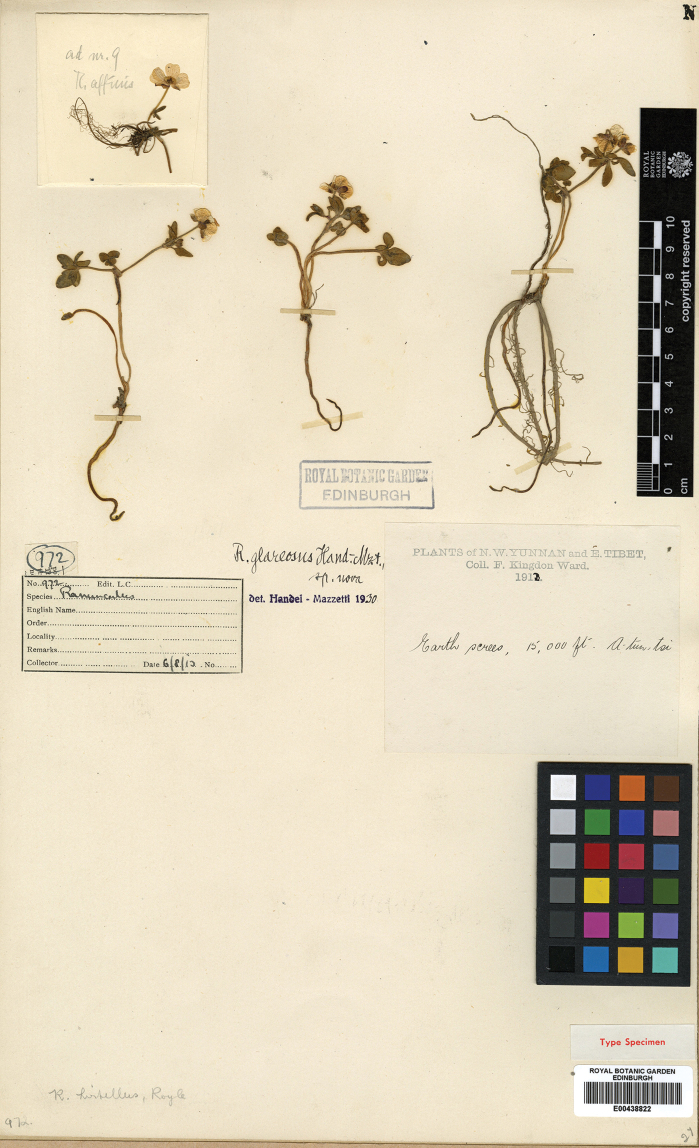
Type sheet of *Ranunculusglareosus*.

**Figure 12.**
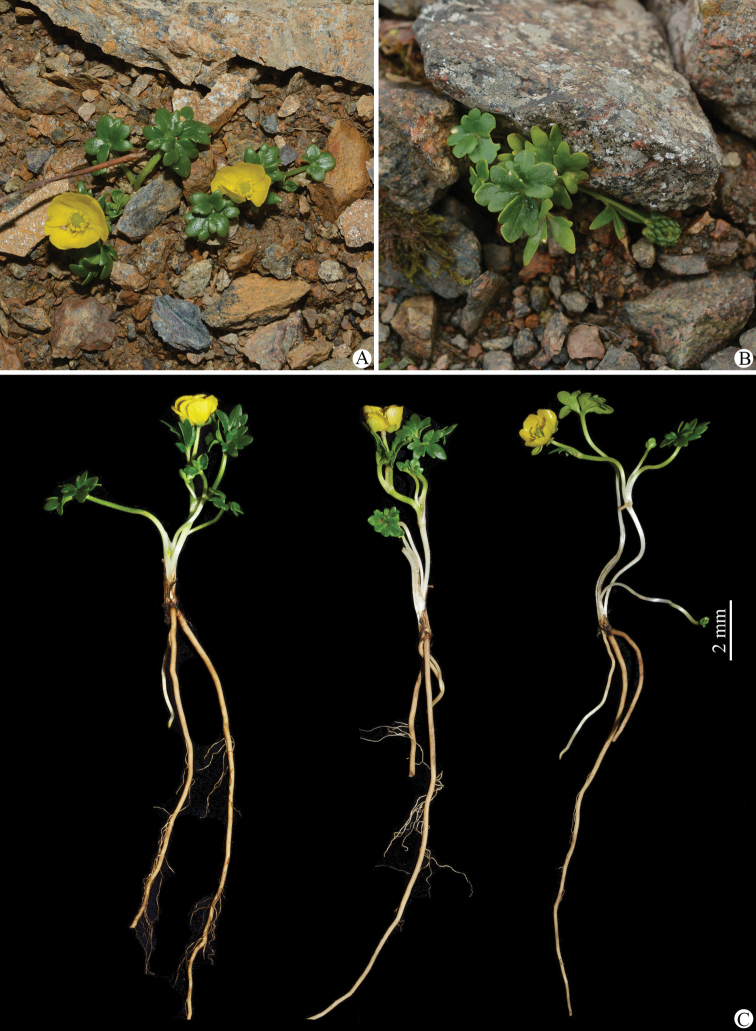
*Ranunculusglareosus* in the wild (China, Qinghai, Menyuan) **A, B** habitat **C** habit. Photographed by Wen-Qun Fei.

**Figure 13.**
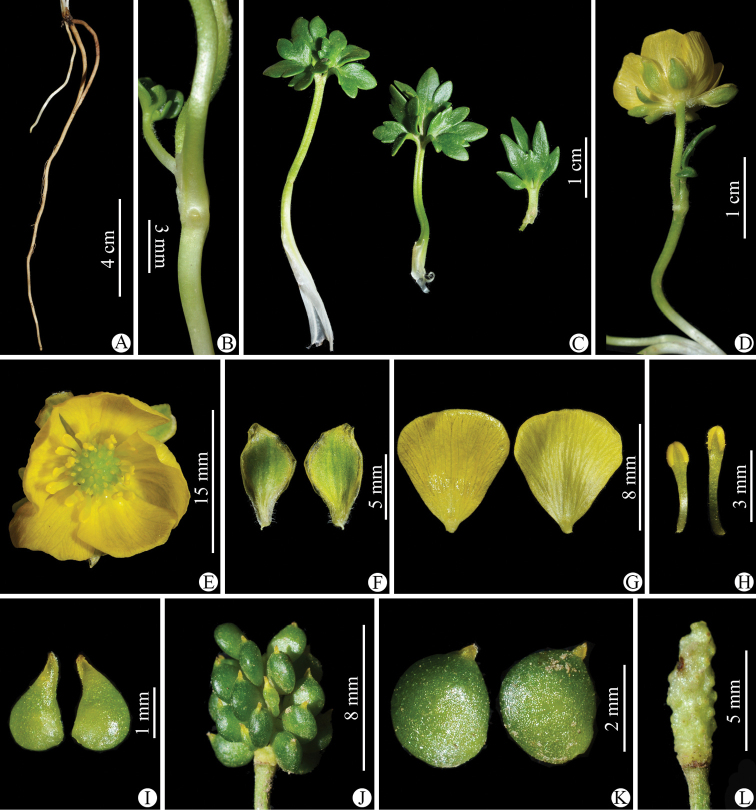
*Ranunculusglareosus* in the wild (China, Qinghai, Menyuan) **A** roots **B** portion of stem **C** leaves **D** flower (lateral view) **E** flower (top view) **F** sepal (left: abaxial side; right: adaxial side) **G** petal (left: adaxial side; right: abaxial side) **H** stamens **I** carpels **J** aggregate fruit **K** achenes **L** receptacle. Photographed by Wen-Qun Fei.

**Figure 14.**
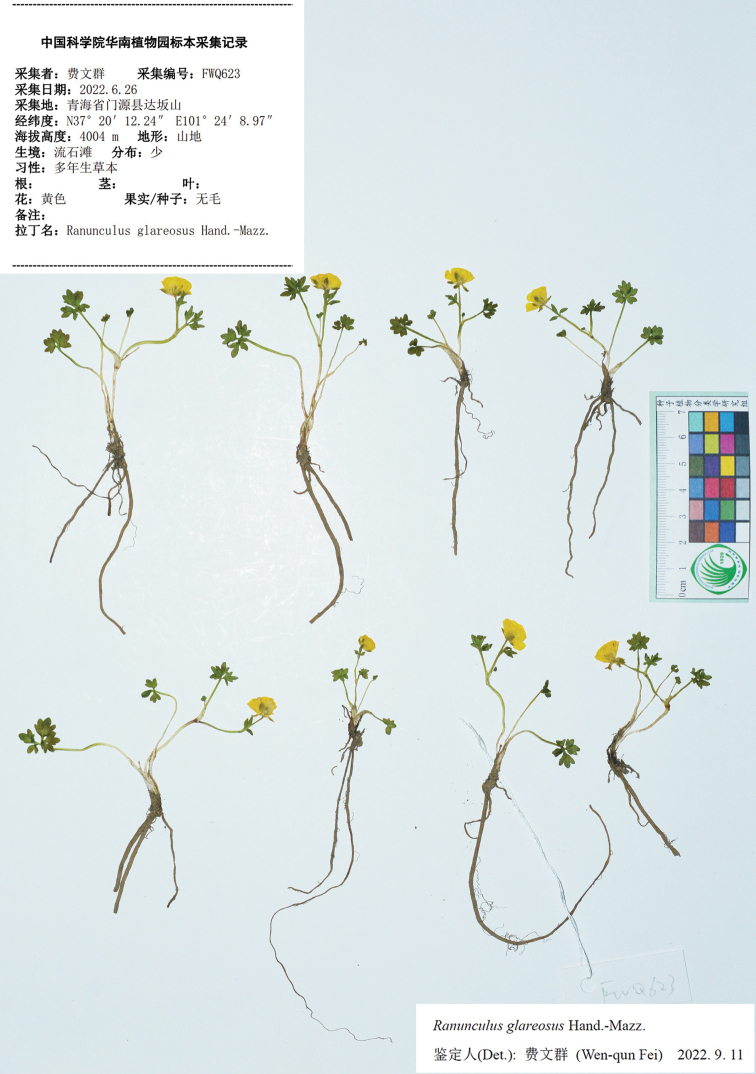
*Ranunculusglareosus*. China, Qinghai, Menyuan, *W.Q. Fei 623* (IBSC).

As mentioned earlier, a gathering of *Ranunculusjiguanshanicus*, *W.B. Ju*, *L. Zhang & D.K. Chen AZH01290* (CDBI), from Chongzhou in Sichuan, the type locality of this species, had been previously misidentified as *R.glareosus* (Figs 11–14). Morphologically, *R.jiguanshanicus* is very easily distinguishable from *R.glareosus* by having glabrous stems (vs. sparsely puberulous) (Figs 3B, 13B), thinly chartaceous leaves (vs. fleshy), leaflets of the 3-foliolate leaves with obvious petiolules (3–5 mm vs. 0.5–2 mm long), unequally 3-sected, with the ultimate leaflet segments narrowly lanceolate to linear, entire or 1–2-denticulate to 1–2-cleft (vs. 3-sected or 3-foliolate, central segment/leaflet ovate or rhombic, entire or 3-lobed, and lateral segments/leaflets flabellate, unequally 2-partite) (Figs 3C, 13C), smaller flowers (5.2–6 mm vs. 15–17 mm in diameter) (Figs 3D, E, 13D, E), abaxially glabrous sepals (vs. puberulous) (Figs 3F, 13F), smaller (3.2–3.5 × 1.8–2 mm vs. 9–10 × 7–8 mm) and obovate petals (vs. widely obovate) (Figs 3G, 13G), subglobose aggregate fruit (vs. ellipsoid) (Figs 3J, 13J), and longer styles in the carpels and achenes (ca. 0.8 mm vs. ca. 0.2 mm long) (Figs 3I, K, 13I, K). In habitat, *R.jiguanshanicus* grows among moss on rocks or rocky cliffs in moist places in fir forests at altitudes of 2900–3150 m above sea level, whereas *R.glareosus* grows on alpine scree slopes at altitudes of 3900–4800 m above sea level. A detailed morphological comparison between *R.glareosus* and *R.jiguanshanicus* is given in Table 1.

#### Additional specimens examined

**(paratypes).** China. **Sichuan**: Chongzhou, *W.B. Ju*, *L. Zhang & D.K. Chen AZH01290* (CDBI); Dayi, *W.Q. Fei 897* (IBSC).

## Supplementary Material

XML Treatment for Ranunculus jiguanshanicus
